# Blocking ion channels induced by antifungal lipopeptide syringomycin E with amide-linked local anesthetics

**DOI:** 10.1038/s41598-018-30077-6

**Published:** 2018-08-01

**Authors:** Anastasiia A. Zakharova, Svetlana S. Efimova, Ludmila V. Schagina, Valery V. Malev, Olga S. Ostroumova

**Affiliations:** 1 Institute of Cytology of the Russian Academy of Sciences, 4 Tikhoretsky prospect, St. Petersburg, 194064 Russia; 2 Saint Petersburg State University, Institute of Chemistry, 26 Universitetskii prospect, St. Petersburg, Petergof 198504 Russia

## Abstract

The effects of the amide-linked (lidocaine (LDC), mepivacaine (MPV), prilocaine (PLC)) and ester-bound local anesthetics (benzocaine (BZC), procaine (PRC), and tetracaine (TTC)) on the pore-forming activity of the antifungal lipopeptide syringomycin E (SRE) in lipid bilayers were studied. Independently on electrolyte concentration in the membrane bathing solution the observed changes in conductance of SRE channels agreed with the altered membrane dipole potential under the action of ester-bound local anesthetics. Effects of aminoamides in diluted and concentrated solutions were completely different. At 0.1 M KCl (pH 7.4) the effects of amide-linked anesthetics were in accordance with changes in the membrane surface potential, while at 2 M KCl aminoamides blocked ion passage through the SRE channels, leading to sharp reductions in pore conductance at negative voltages and 100-fold decreases in the channel lifetimes. The effects were not practically influenced by the membrane lipid composition. The interaction cooperativity implied the existence of specific binding sites for amide-bound anesthetics in SRE channels.

## Introduction

**Figure 1:**
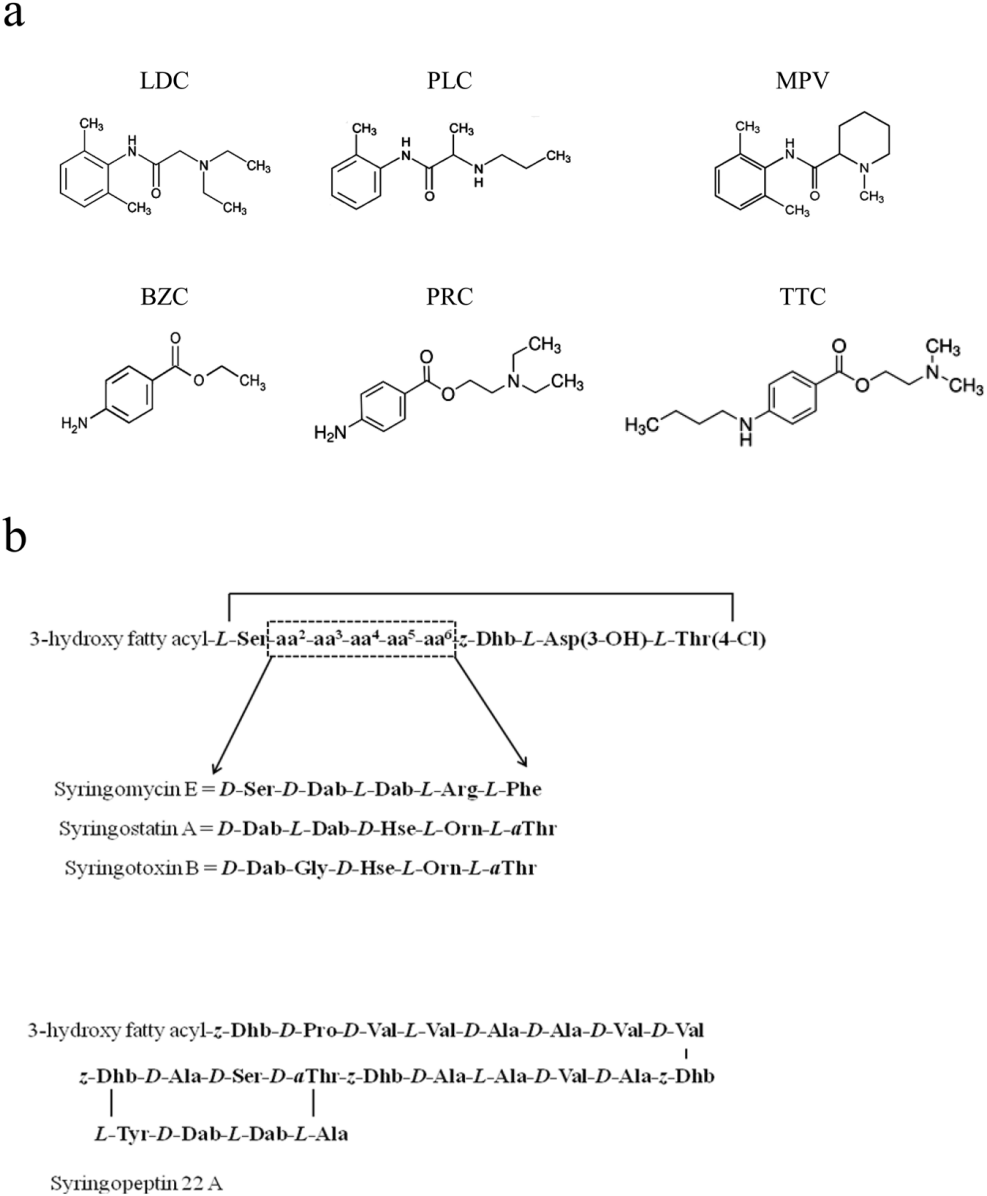
(**a**) Chemical structures of local anesthetics (LDC, PLC, MPV, PRC, BZC, and TTC). (**b**) Structures of tested lipopeptides (SRE, syringostatin A, syringotoxin B, and syringopeptin 22 A). The first four lipopeptides differ in the amino acid sequence between positions 2 and 6. The 3-hydroxy fatty acyl group is a derivative of decane, dodecanoic, tetra- or hexadecanoic acid. Abbreviations for nonproteinogenic amino acids: Asp(3-OH) – 3-hydroxyaspartic acid; Dab – 2,4-diaminobutyric acid; Dhb – 2,3-dehydroaminobutyric acid; Hse – homoserine; Orn – ornithine; Thr(4-Chl) – 4-chlorothreonine; *a*Thr – *allo*threonine.

Local anesthetics are compounds causing the suspension of impulse transmission along nerve fibers, and their molecular structure comprises hydrophobic and lipophilic parts connected by amide or ether bonds. Correspondingly, they are divided into amide-linked (e.g., LDC, MPV, and PLC) and ester-bound (e.g., BZC, PRC, and TTC) groups. The chemical structures of local anesthetics are presented on the Fig. 1a. As a rule, at physiological pH, these drugs exist in both neutral and cationic forms. Among the listed anesthetics, only BZC is excluded, which is almost completely uncharged under physiological conditions.

Local anesthetics interfere with impulse conductions in nerves and muscles by binding to voltage-gated sodium channels and blocking the transient Na^+^ inward current^1^. While the major drug mechanism of action is not resolved, experimental evidence favors steric blocking, closed state stabilization, or some combination of the two. Extensive site-directed mutagenesis experiments have provided proof that LDC-like drugs bind in the inner pore^2–4^. However, substantial evidence supports that anesthetics generally perturb bulk membrane structure^5–7^ and, consequently, affect membrane transport, especially ion channels^8–10^. Specifically, a good correlation between the partition coefficients in lipid/water systems (and, consequently, their ability to rearrange membrane lipids) and the clinical potency of the drugs support the hypothesis that membrane lipids are the primary sites of anesthetic action^11,12^. Lee *et al*.^8^ proposed that local anesthetics trigger the transition of surrounding lipids to the more fluid, liquid crystalline phase, allowing the sodium channel to close, resulting in local anesthesia.

Since anesthetics are present in both cationic and non-ionic forms under physiological conditions, the question of whether uncharged or charged species affect the physical properties of the membrane has been raised. Fraceto *et al*.^13^ showed that the uncharged versions of LDC and MPV increase the mobility of choline nuclei and decrease the mobility of glycerol-region hydrogens, thus modulating lipid packing. Hata *et al*.^14,15^ studied the effects of local anesthetics on the phase transition temperatures of dipalmitoylphosphatidylcholine bilayer membranes by optical and calorimetrical methods. TTC, LDC and PRC depressed the main and pre-transition temperatures of the DPPC bilayers. Moreover, TTC induced complex phase behaviors of dipalmitoylphosphatidylcholine, including the formation of mixed lipid/anesthetic micelles and transition from the interdigated gel phase (rather than the ripple phase) to the liquid crystalline phase at relatively high TTC concentrations. Using high-pressure Fourier transform infrared spectroscopy, Auger *et al*.^16^ demonstrated smaller interchain interactions for dimyristoylphosphatidylcholine due to increases in both orientational and conformational disorder caused by uncharged TTC intercalation between lipid acyl chains. The authors concluded that uncharged TTC disorders myristoyl chains, while the charged form induces the formation of an interdigitated gel phase. The authors also showed that cholesterol (CHOL) prevents the formation of the interdigitated phase.

In addition to modifying the elastic properties of membranes, anesthetics electrostatically interact with the lipid bilayer, i.e., the drugs alter the electrical potential at the water/lipid boundary^17^, termed the membrane boundary potential. The boundary potential consists of two components^18–23^: a surface component (φ_s_), related to the surface charge of the membrane, and the dipole component (φ_d_), due to specifically orientated lipid and water dipoles at the interface, which imparts a highly positive electrostatic potential to the membrane interior membrane in respect to the adjacent aqueous phase and, consequently, regulation of reconstituted ion channels^24–35^. Furthermore, TTC was found to neutralize the negative surface charges of cardiolipin-containing liposomes^36^. The authors also concluded that TTC increases surface potential more effectively than LDC^37^. Furthermore, both the charged and uncharged forms of TTC and LDC induce substantial changes in the membrane dipole potential^38,39^.

This study aimed to establish the mechanism underlying the influence of local anesthetics on SYRingomycin E channels. The antifungal lipopeptide SRE was previously shown to form voltage-gated, predominantly anion-selective asymmetric cone-shaped channels with a narrow peptide and a wide lipid mouth^40^. The topology of SRE channel water pores is like that of sodium channels in open state, with a relatively wide vestibule and a narrower region, including the selectivity filter^41^. Moreover, SRE channels are characterized by potential and mechanical sensitivity. Altogether, these facts make SRE channels extremely convenient for studying the binding and lipid-mediated effects of local anesthetics. To test the possibility of binding, we used the drugs of two types, amide-linked (LDC, PLC, and MPV) and ester-bound anesthetics (BZC, PRC, and TTC). Binding cooperativities were evaluated by Hill’s coefficients^42^. Changes in the cation/anion selectivities of the channels are thought to be determined by the binding anesthetics in a narrow part of the pore. To establish whether the lipid-mediated effects of the drugs are caused by alterations in the surface or dipole components of the membrane boundary potential, two different concentrations of an electrolyte in bilayer bathing solutions were tested (0.1 and 2 M KCl). To identify the possible actions of local anesthetics on lipid packing, pure bilayers from dioleoylphosphocholine (DOPC) were compared with CHOL enriched membranes. Lipid bilayers comprising anionic phospholipids, dioleoylphosphoserine (DOPS), and raft-mimicking mixture of DOPC, sphingomyelin (SM), and CHOL were also tested.

## Results and Discussion

**Figure 2:**
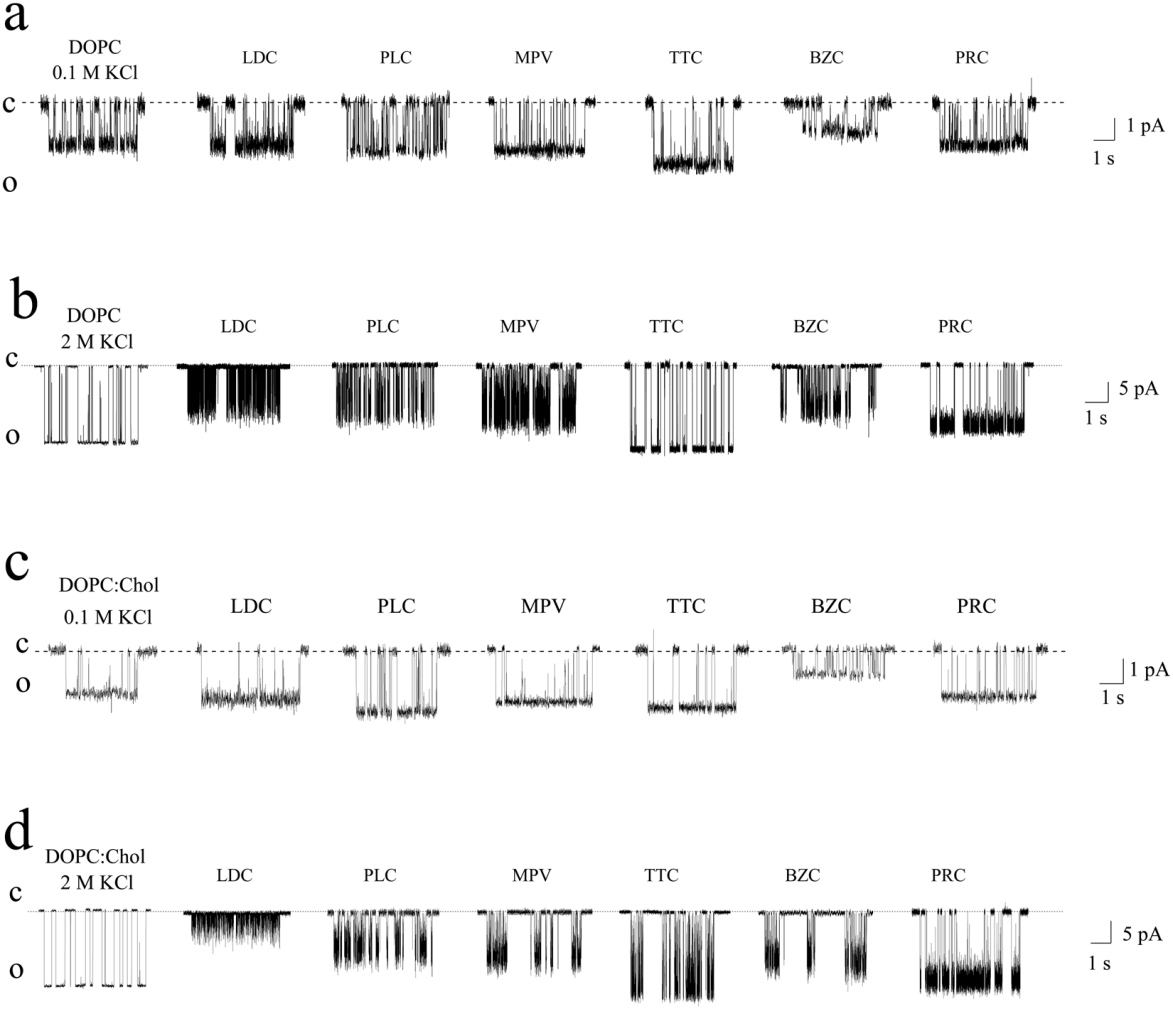
Current fluctuations corresponding to the openings and closings of single SRE channels in lipid bilayers in the absence (*control*) and presence of various local anesthetics: 10 mM LDC, PLC, MPV and PRC; 3 mM BZC; and 1 mM TTC. The membranes were composed of DOPC (**a**,**b**) or DOPC:CHOL (67:33 mol%) (**c**,**d**) and bathed in 0.1 M (**a**,**c**) or 2.0 M KCl (pH 7.4) (**b**,**d**). The transmembrane voltage was equal to −150 mV. *C* – closed state of the channel, *O* – open state of the pore.

**Figure 3:**
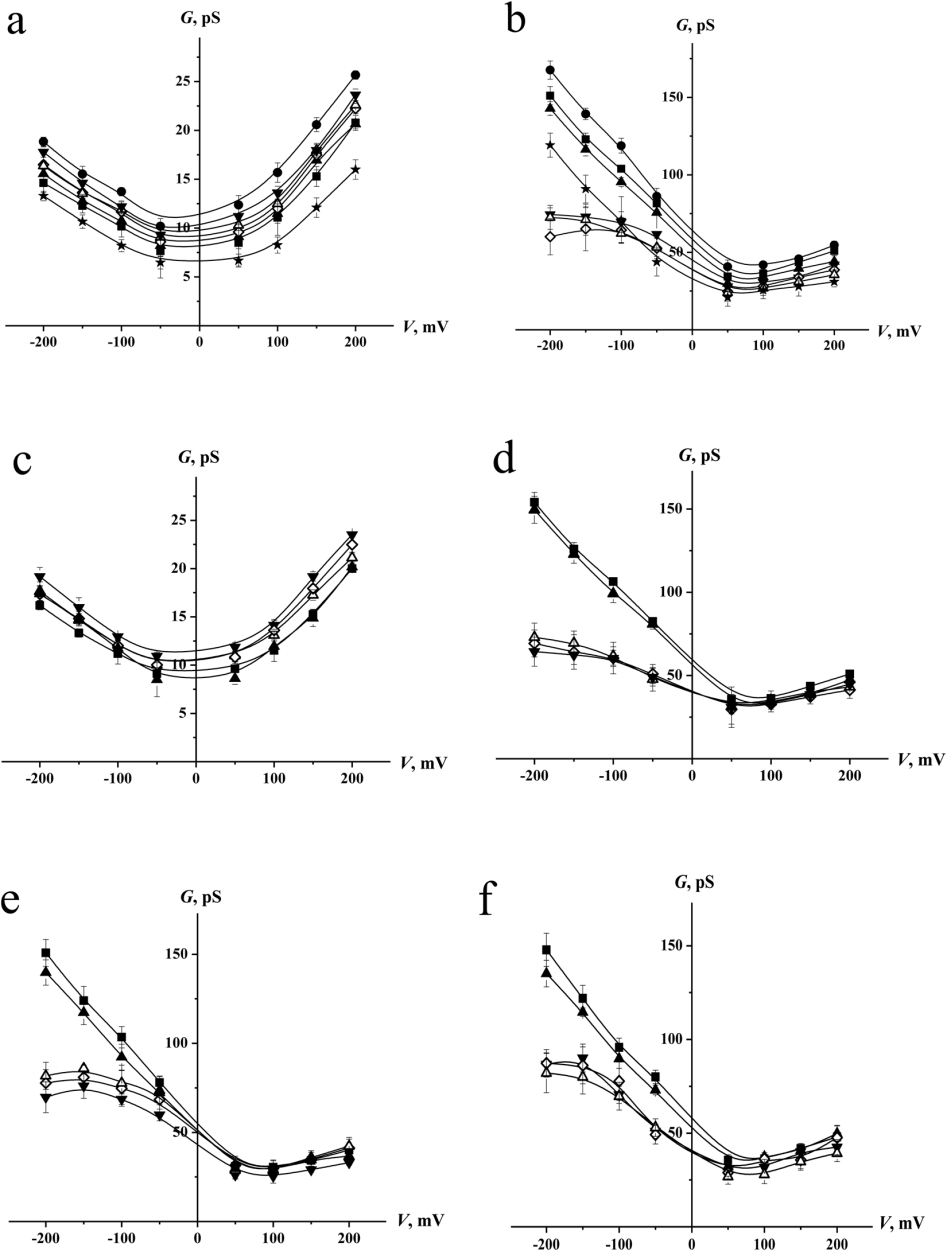
*G*(*V*) curves of single SRE channels in the absence (■) and presence of 10 mM LDC (◊), PLC (▼), MPV (Δ) or PRC (▲); 3 mM BZC (*); and 1 mM TTC (●). The membranes were composed of DOPC (**a**,**b**), DOPC:CHOL (67:33 mol%) (**c**,**d**), DOPS (**e**) or DOPC:CHOL:SM (47:33:20 mol %) (**f**) and bathed in 0.1 M (**a**,**c**) or 2.0 M KCl (pH 7.4) (**b**,**d**,**e**,**f**).

Figure 2a demonstrates the current fluctuations corresponding to the openings and closings of single SRE channels in DOPC membranes bathed in 0.1 M KCl (pH 7.4) in the absence (control) and presence of 10 mM LDC, PLC, MPV, and PRC; 3 mM BZC; and 1 mM TTC. LDC, PLC, MPV, and TTC enhanced the amplitude of the SRE channels, while PRC did not influence this value. BZC significantly reduced the channel conductance. Figure 3a shows the corresponding *G*(*V*) curves in the absence and presence of the local anesthetics. LDC and MPV increased *G* by approximately 15%, while PLC and TTC enhanced *G* by approximately 30%. Simultaneously, PRC did not change *G*, while BZC reduces the channel amplitude by approximately 20%. We previously showed that local anesthetics affect the boundary potential of model lipid membranes. Changes in the boundary potential by the positively charged amide-linked anesthetics LDC, PLC, and MPV are due to increases in the surface potential of the bilayer, while TTC increases its dipole potential^17^. Indeed, the surface potential increment of the DOPC membrane at 10 mM LDC, PLC and MPV was approximately 24 ± 5 mV. The increase in the dipole potential of DOPC bilayers induced by 1 mM TTC equaled 48 ± 14 mV. PRC did not obviously affect the magnitudes of the surface and dipole components of the DOPC membrane boundary potential at concentrations up to 10 mM. Here, we evaluated the changes in dipole potential induced by the addition of 3 mM BZC to the bathing solution, which reduced the dipole potential by 55 ± 8 mV. Comparing the effects of the drugs on the SRE channel current amplitude and electrical potential at the membrane/water interface, the changes in *G* upon the introduction of anesthetics presumably correspond to the changes in the boundary potential, its dipole or surface components.

To verify the above assumption, *G* was measured at a high electrolyte concentration in the membrane bathing solution (2 M KCl at pH 7.4), i.e., under the condition of screening the surface potential by electrolyte ions. In this case, only the effects of the membrane dipole potential should be observed. Figure 2b presents the current fluctuations of single SRE channels in DOPC membranes bathed in 2 M KCl (pH 7.4) in the absence and presence of the local anesthetics. TTC increased *G*, BZC noticeably reduced *G*, and PRC did not obviously change *G*. Figure 3b demonstrates the corresponding *G*(*V*) curves before and after the addition of the drugs. The observed differences in shapes of the conductance-voltage characteristics of SRE channels in dilute and concentrated electrolyte solutions were discussed in^43^. TTC increased *G* by 15%, while BZC reduced the channel amplitude by 30%. The ester-bound anesthetics did not affect the shape of the *G*(*V*) curve. Thus, the effects of TTC, PRC, and BZC on *G* at 0.1 and 2 M KCl are the same and, for this reason, can be attributed to the altered dipole potential. Surprisingly, at 2 M KCl, 10 mM LDC, PLC and MPV pronouncedly reduced the SRE channel conductance at negative voltages. As indicated in the Materials and methods section, negative voltage signs are related to the flow of cations from the *trans* to the *cis* side of the experimental chamber. Considering that SRE channels are asymmetric, cone-shaped peptide-lipid pores, with the *cis* opening being much narrower than the *trans* opening^41^, cations of amide-linked anesthetics presumably penetrate the SRE pore from its wider *trans* mouth, which leads to reductions in *G* at negative voltages. The electrostatic repulsion between positively charged molecules of amide-bound local anesthetics and SRE prevented the permeation of LDC, PLC and MPV into the channel at low electrolyte concentrations in the membrane bathing solution. Thus, at high electrolyte concentrations with smaller Debye radii, steric blocking of the SRE channel by amide-linked anesthetics might occur.

The blocking of SRE channels by anesthetics should primarily result in decreased channel opening times. To verify this assumption, the dwell times of SRE channels were measured. Table 1 shows that the effects of the amide-bound drugs on the SRE channels at low and high salt concentrations were not the same. These compounds drastically decreased *τ* at 2 M KCl (up to 20-fold) compared to the slight reduction observed at 0.1 M KCl. The ester-linked anesthetics TTC and BZC reduced *τ* several fold at both 0.1 and 2 M KCl, while PRC did not obviously change this value.

**Table 1:** The mean dwell times (*τ*, ms*) and transfer numbers of anions (*t*^*−*^) of single SRE channels in the absence (control) and presence of various local anesthetics. The membranes were composed of DOPC, DOPC:CHOL (67:33 mol%), DOPC:CHOL:SM (47:33:20 mol%), or DOPS and bathed in 0.1 M KCl or 2.0 M KCl (pH 7.4).

Anesthetic	*C*, mM	ρ, %# at pH 7.4	*τ*, ms*						t^−&^
			DOPC		DOPC:CHOL		DOPC:CHOL:SM	DOPS	
			0.1 M KCl	2.0 M KCl	0.1 M KCl	2.0 M KCl	2.0 M KCl	2.0 M KCl	
*control*	—	—	187 ± 3	74 ± 1	280 ± 6	253 ± 4	123 ± 3	198 ± 5	0.86 ± 0.07
*LDC*	10	75	152 ± 15	1 ± 1	161 ± 9	1 ± 1	1 ± 1	3 ± 1	0.53 ± 0.21
*PLC*		76	128 ± 12	16 ± 1	141 ± 3	13 ± 1	9 ± 1	15 ± 1	0.61 ± 0.09
*MPV*		64	152 ± 4	11 ± 1	207 ± 21	8 ± 1	2 ± 1	14 ± 1	0.75 ± 0.04
*BZC*	3^$^	<1	63 ± 2	22 ± 1	30 ± 1	26 ± 1	—	—	0.78 ± 0.04
*PRC*	10	99	192 ± 14	110 ± 3	245 ± 8	115 ± 2	128 ± 9	146 ± 6	0.78 ± 0.05
*TTC*	1^$^	97	49 ± 1	57 ± 1	121 ± 3	48 ± 3	—	—	0.89 ± 0.06

^$^BZC and TTC are not able to be measured at 10 mM due to lower water/lipid solubility.

*The values of dwell time were averaged by the voltage range from −200 to 200 mV.

^#^The portion of molecules of local anesthetics in a charged form at pH 7.4 was calculated using the Henderson–Hasselbalch equation.

^&^The transfer numbers of anions through the single SRE channels was measured in DOPC membranes in a 10-fold salt gradient between the *cis* and *trans* chamber compartments (0.4 M KCl/4 M KCl in the presence of 10 mM LDC, PLC, MPV, PRC, and 0.2 M KCl/2 M KCl in the presence of 3 mM BZC or1 mM TTC).

The slight reductions in SRE channel opening times caused by anesthetics of both types at 0.1 M KCl and by ester-linked compounds at 2 M KCl might be attributed to the generally recognized disordering effect of local anesthetics on the lipid bilayer^6–10,12^ and/or their influence on a distribution of lateral pressure in a membrane^44^. CHOL is known to play a key role in controlling membrane fluidity, and the incorporation of CHOL into the membrane leads to the increased ordering of lipid hydrocarbon chains and a reduction in the area per molecule^45,46^. The current fluctuations of single SRE channels in DOPC membranes enriched with CHOL (33 mol%) and bathed in 0.1 and 2 M KCl in the absence (control) or presence of the drugs are shown in Fig. 2c,d, respectively. Figure 3c,d show the corresponding *G*(*V*) curves in the absence and presence of the amide-bound anesthetics and procaine. Table 1 shows that in the absence of anesthetics, the introduction of CHOL into the membrane forming solution increased *τ* at both 0.1 M and 2 M KCl. As expected, CHOL contributes to the deoligomerization of SRE molecules during pore dismantling. Table 1 shows that the effects of anesthetics on *τ* in CHOL-free and CHOL-enriched lipid bilayers are similar. Thus, the observed effects of the ester-linked anesthetics at both 0.1 M KCl and 2 M KCl and the amide-bound compounds at 0.1 M KCl on τ are most likely related to membrane disordering. Therefore, significant decreases in *τ* induced by amide-linked anesthetics might be due to the pore blocking by local anesthetics at 2 M KCl.

**Figure 4:**
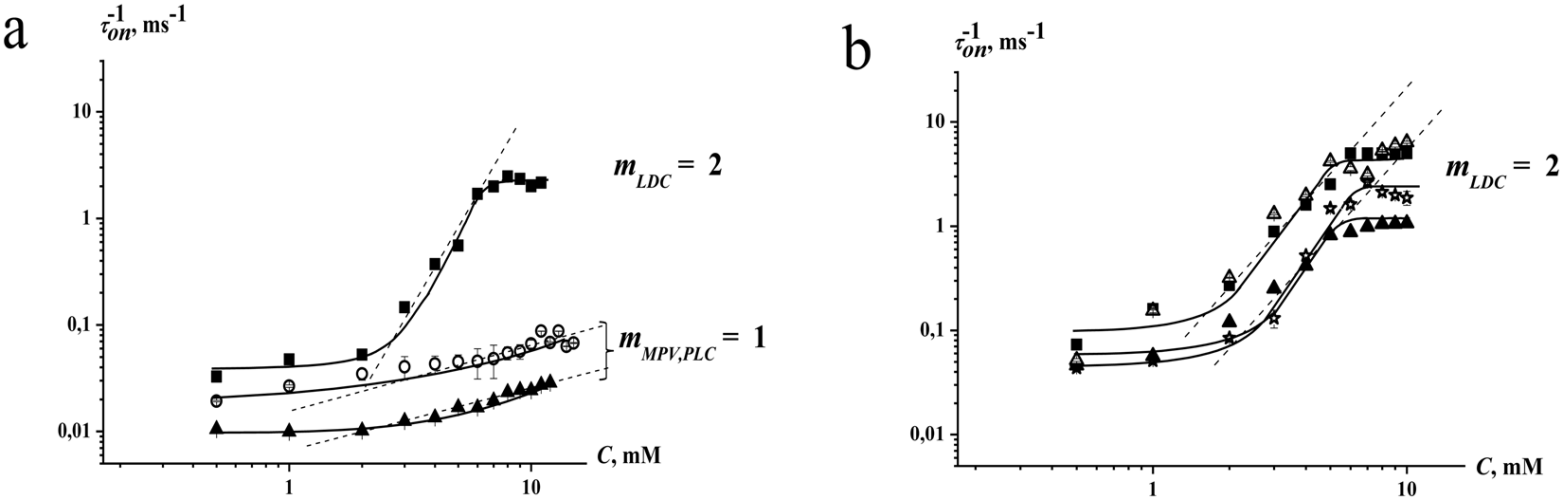
(**a**) Dependence of the inverse mean dwell times of SRE channels (τ_on_^−1^) on the concentrations (*C*) of LDC (■), PLC (▲), and MPV (○). (**b**) Dependence of the inverse mean dwell times of syringopeptin 22 A (□), syringostatin A (*), syringotoxin B (▲), and CH3-SRE (∆) channels on the concentration of LDC. The straight lines represent the linear approximations of the dependence growth linear regions. The slopes of the lines characterize the numbers of anesthetic molecules interacting with single channels formed by different lipopeptides (*m*). The membranes were composed of DOPC and bathed in 0.1 M KCl (pH 7.4). The transmembrane voltage was equal to −100 mV.

It is known that anesthetics show a preference for specific membrane domains, namely the lipid rafts^47^, and their membrane-fluidizing effects might be influenced by anionic phospholipids^48^. In order to further examine the assumption that the observed effects of amide-bound drugs on SRE channels do not qualitatively depend on membrane lipid nature, we additionally tested bilayers comprising of pure DOPS (Fig. 4e) and DOPC:CHOL:SM (47:33:20 mol %) (Fig. 4f) at 2 M KCl. Comparing Fig. 4b,d,e,f one can conclude that the effects of amide-linked anesthetics on *G* are not practically influenced by the membrane lipid composition, in particular, by the presence of anionic lipids or lipid ordered domains enriched with cholesterol and SM. The action of the anesthetics on *τ* in bilayers comprising of DOPC, DOPS, DOPC:CHOL and DOPC:CHOL:SM are also similar (Table 1). These data are consistent with the hypothesis of direct blocking SRE pore with the anesthetics.

Using Hyperchem 7.0, we evaluated the mean sizes of the anesthetic molecules, corresponding to their larger linear dimensions. Thus, we approximated the LDC, MPV, PLC, PRC, TTC and BZC molecules by spheres with diameters of 10.3, 7.8, 10.8, 13.4, 16.9, and 10.4 Å (sd = 0.1), respectively. Considering that the mean diameter of the wider lipidic mouth of an SRE channel is approximately 14 Å^41^, LDC, MPV, PLC, and BZC potentially permeate the SRE pore from the *trans* side, while PRC and TTC penetration is restricted. The amide-bound drugs have positive charges at neutral pH and can penetrate the SRE pore only at high electrolyte concentrations due to small Debye lengths. BZC is not charged at neutral pH and can reduce *G* at both 0.1 M and 2 M KCl (Fig. 3a,b, respectively) due to the partial occupation of the pore volume by non-electrolytes.

To evaluate the stoichiometry of blocking the SRE channel with the amide-linked anesthetics, we studied the dependence of *τ* on the anesthetic concentration (Fig. 4). The dependences are presented in two logarithmic coordinates; in this case, the linear approximation of *τ*(*C*) curve growth regions gave the Hill’s coefficients^42^ for the analysis of anesthetic cooperative binding with the SRE channel. The coefficients were equal to 1 for MPV and PLC, while that for LDC was approximately 2 (Fig. 4a). Thus, the cooperativity of the interaction clearly indicates the existence of specific binding sites for amide-bound anesthetics in SRE channels. Suspecting its aromatic nature, as is the case for voltage-dependent sodium channels, we tested the structurally similar lipopeptides syringotoxin B, syringostatin A, and syringopeptin 22 A and the Asp-methylated form of SRE (CH_3_-SRE). Unlike SRE, CH_3_-SRE, and syringopeptin 22 A, syringotoxin B, and syringostatin A do not have any aromatic amino acids in their peptide heads (Fig. 1b). Figure 4b presents the dependence of the inverse mean dwell times of channels formed by CH_3_-SRE, syringotoxin B, syringostatin A, and syringopeptin 22 A on the LDC concentration in the two logarithmic coordinates. Similar to the SRE pore, two LDC molecules bound single channels formed by all of the tested lipopeptides. Considering that several (about 6) lipopeptide molecules form single conductive unit^49^ and comparing the structures of the pore-forming compounds, we proposed that dehydroaminobutyric acid residues might be the binding sites because of double bonds in their side chains.

**Figure 5:**
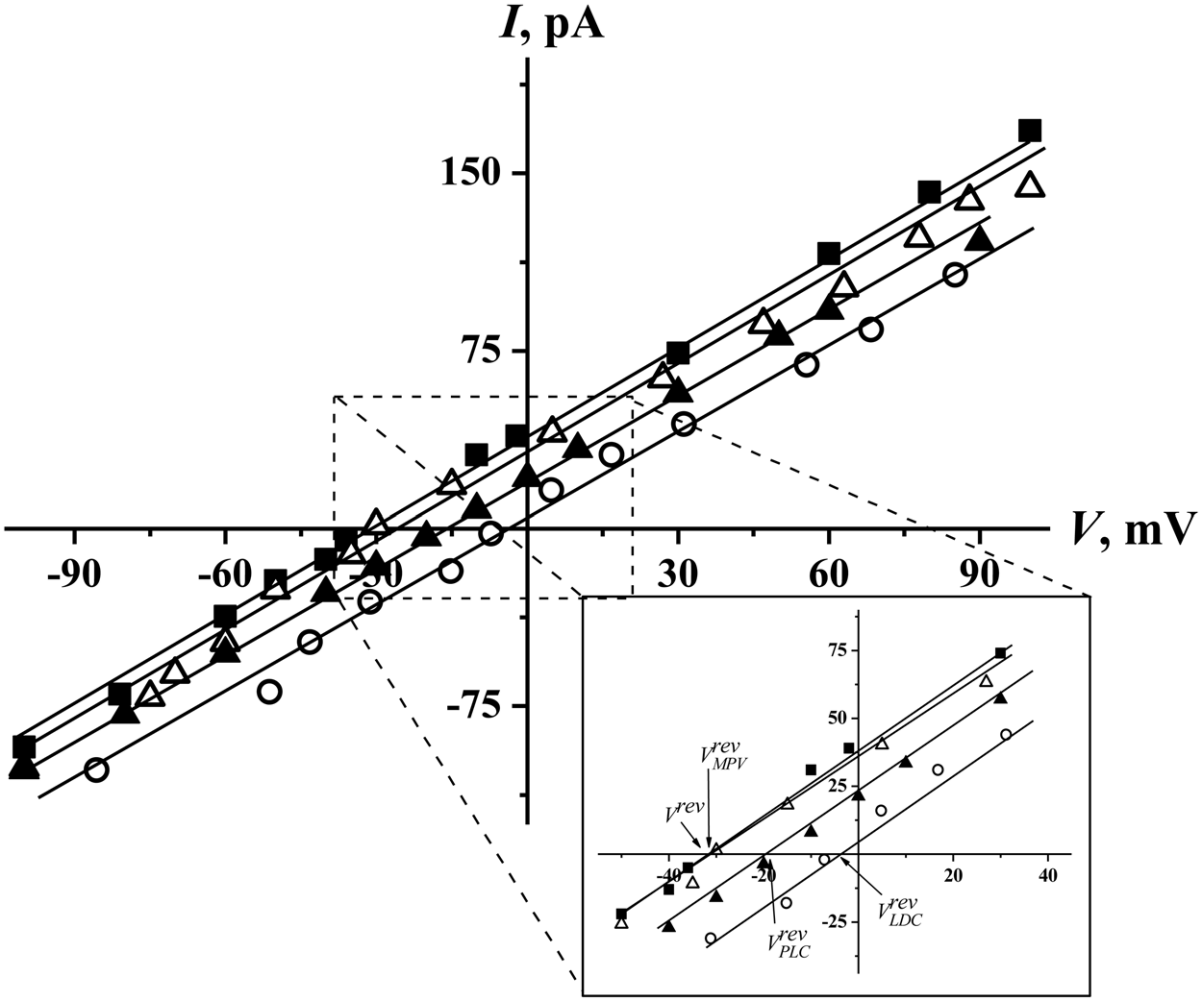
Anion/cation selectivities of single SRE channels in DOPC membranes*. I*(*V*) curves of bilayers treated with SRE in the absence of the drugs (■) and presence of 10 mM LDC (○), PLC (▲), and MPV (∆) in an asymmetric electrolyte concentration system. The 0.4 M KCl solution (pH 7.4) comprised the *cis* compartment, while the 4 M KCl solution (pH 7.4) comprised the *trans* compartment. *Inset*: the intercepts on the *V* axis represent the reversal potentials in the absence (*V*^*rev*^*)* and presence of LDC (*V*_*LDC*_^*rev*^*)*, PLC (*V*_*PLC*_^*rev*^), and MPV (*V*_*MPV*_^*rev*^).

**Figure 6:**
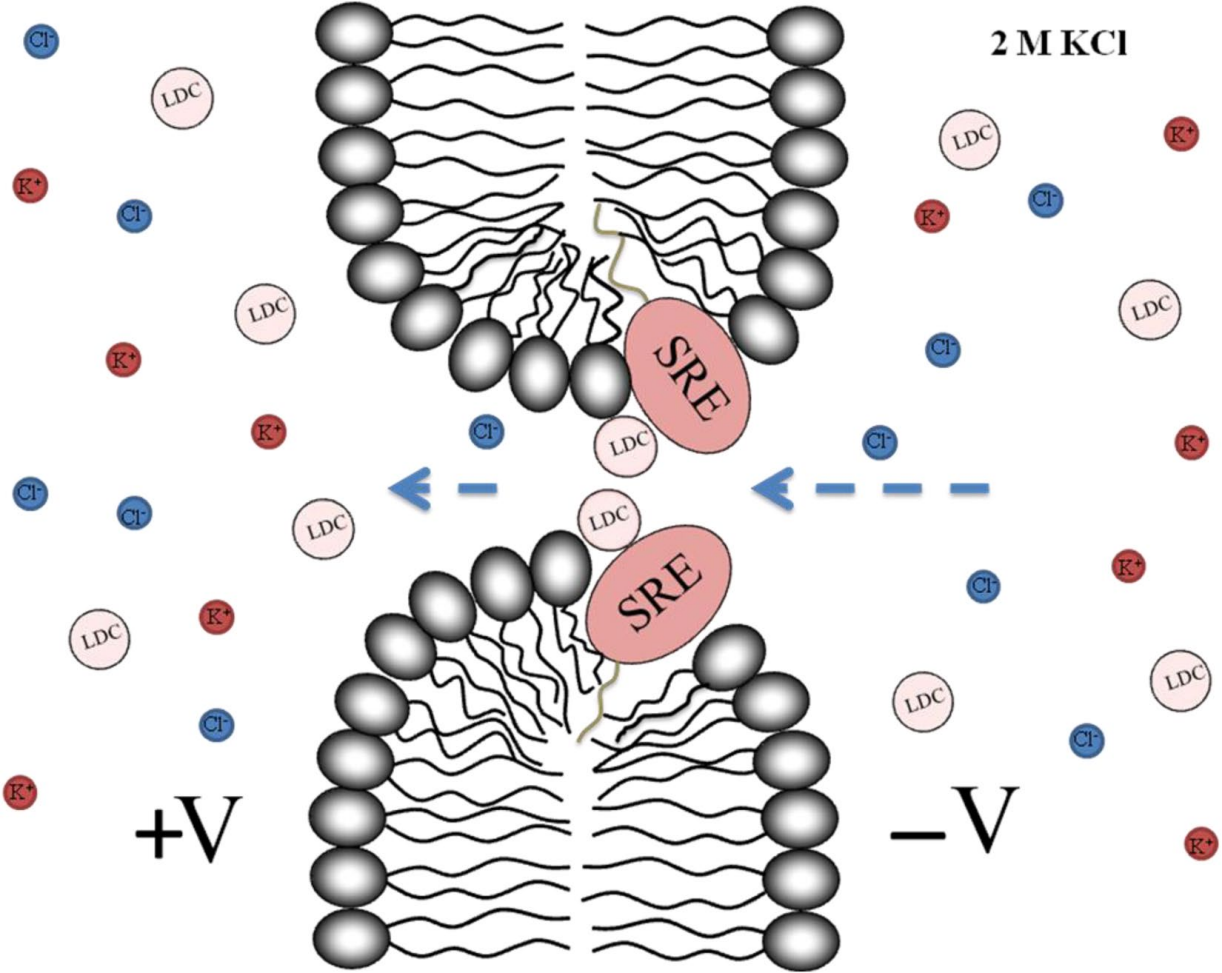
Schematic representation of SRE channel blocking with LDC.

The specific binding of positively charged anesthetics in the narrow, lipopeptide part of the channel should also influence pore selectivity. Figure 5 demonstrates the different cation/anion selectivities of the bilayers treated with SRE in the absence and presence of amide-bound drugs. Before the addition of the anesthetics to the membrane bathing solution at a 10-fold electrolyte gradient through the membrane, the reversal potential was −40 ± 6 mV, while the values of *V*^*rev*^ in the presence of LDC, MPV, and PLC were equal to −4 ± 4, −28 ± 2, and −13 ± 9 mV, respectively. The reversal potential in the absence of the drugs corresponded to the anion transfer number, equaling 0.86 ± 0.07 (Table 1). The similar values of the transfer numbers were obtained in the presence of ester-bound anesthetics. The anion transfer numbers in the presence of 10 mM LDC, MPV, and PLC were 0.53 ± 0.21, 0.75 ± 0.04, and 0.61 ± 0.09, respectively. Thus, the data obtained agree with the assumption that the blocking of SRE channels with amide-bound anesthetics allows their interaction with peptide part similar to those reported for channel proteins^1^ and unlike the blocking of spontaneous lipid pores with anesthetics^50^. Figure 6 schematically represents SRE channel blocking with LDC.

## Conclusions

Based on the above considerations, the amide-linked anesthetics LDC, MPV, and PLC block ion passage through SRE channels at high electrolyte concentrations in the membrane bathing solution. The local anesthetics permeate the SRE pore from its wider *trans* mouth, which leads to a sharp drop in channel conductance at negative voltages and a decrease in its lifetime by two orders of magnitude. The interaction cooperativity observed with LDC indicates the existence of specific binding sites for amide-bound anesthetics in SRE channels, and possible binding site candidates include dehydroaminobutyric acid residues. The ester-bound anesthetics TTC and BZC affect SRE channel conductance in accordance with changes in the bilayer dipole potential, while alterations in the pore dwell times are related to the disordering effects of anesthetics on lipid bilayers. The action of BZC, which was neutral at the pH used, might include the reduction in channel amplitude due to the permeation of non-electrolytes into the SRE pore.

## Materials and Methods

All of the chemicals used in this study were of analytical grade. DOPC, DOPS, SM and CHOL were obtained from Avanti Polar Lipids, Inc. (Pelham, AL). The local anesthetics (BZC and hydrochlorides of LDC, PLC, MPV, PRC, and TTC), KCl, HEPES, and hexadecane were purchased from Sigma Chemical (St. Louis, MO). Water was distilled twice, deionized and degassed.

SRE, syringopeptin 22 A, syringostatin A, and syringotoxin B were isolated and purified as described previously^51^ and kindly provided by Dr. J.Y. Takemoto (Utah State University, USA). CH_3_-SRE was synthesized in the lab of Dr. J.Y. Takemoto.

### Formation of planar lipid bilayers and registration of single channels

Virtually solvent-free planar lipid bilayers were prepared using a monolayer-opposition technique^52^ on a 50-μm-diameter aperture in a 10-μm-thick Teflon film separating two (*cis* and *trans*) compartments of a Teflon chamber. The aperture was pretreated with hexadecane. Lipid bilayers were made from DOPC, DOPS, and mixtures of DOPC:CHOL (67:33 mol%), and DOPC:CHOL:SM (47:33:20 mol %). Solutions of 0.1 M or 2.0 M KCl were the same in both Teflon chamber compartments and were buffered by 5 mM HEPES-KOH at pH 7.4. After the membrane was completely formed and stabilized, liopeptides from a stock solution (5 mM in water, pH 3.0) were added to the aqueous phase at *cis* side of the bilayer to obtain a final concentration ranging from 1 to 5 μM for SRE, 0.1 ÷ 0.2 μM for syringopeptin 22 A, 1 ÷ 2 μM for syringostatin A, 3 ÷ 13 μM for syringotoxin B and 4 ÷ 7 μM for CH_3_-SRE. Ag/AgCl electrodes with 1.5% agarose/2 M KCl bridges were used to apply the transmembrane voltage (*V*) and measure the transmembrane current (*I*). “Positive voltage” referred to when the *cis* side compartment was positive relative to the *trans* side compartment.

The anesthetics from a 600 mM water stock solution were added to both sides of the membranes at final concentrations in the range from 0.1 to 10 mM for LDC, PLC, MPV; 10 mM for PRC; 3 mM for BZC; and 1 mM for TTC. The choice of the concentrations used for different anesthetics was determined by their buffer/lipid solubility and the dependences of physical properties of lipid bilayers and reconstituted syringomycin channels on the concentration of tested drugs.

Current measurements were carried out using an Axopatch 200B amplifier (Molecular Devices, LLC, Orlean, CA, USA) in the voltage clamp mode. Data were digitized by Digidata 1440 A and analyzed using pClamp 10 (Molecular Devices, LLC, Orlean, CA, USA) and Origin 7.0 (OriginLab Corporation, Northampton, MA, USA). Data acquisition was performed with a 5-kHz sampling frequency and low-pass filtering at 200 Hz. The current tracks were processed through an 8-pole Bessel 100-kHz filter. Single-channel conductance (*G*) was defined as the ratio between the current flowing through a single SRE channel and the transmembrane voltage. All experiments were performed at room temperature.

The *I* values and channel dwell times (τ) histograms were constructed for the tested voltages. The relative frequency (*n*/*N*) was set as the histogram ordinate, where *n* was the number of current fluctuations corresponding to a given current or time level and *N* was the total number of fluctuations. For conductance fluctuation analysis, *N* ranged from 500 to 2000. Peaks on the *I* histograms were fitted by the normal density function. For the dwell time histograms, *N* was equal to 1000–3000, and the distribution was fitted by an exponential density function. The distribution hypotheses were verified using χ^2^ minimization (*P* < 0.05).

### Estimation of changes in the membrane dipole potential upon BZC adsorption

The steady-state membrane conductance induced by K^+^-nonactin was modulated via the two-sided addition of BZC to the membrane bathing solution up to a final concentration of 3 mM. Lipid bilayers were made from pure DOPC, and 0.1 M solutions were buffered using 5 mM HEPES–KOH at pH 7.4. Considering that BZC is neutral at pH 7.4, changes in the electrical potential at the membrane/water boundary should be attributed to only changes in the membrane dipole potential (∆φ_d_), and this value can be evaluated assuming that membrane conductance is related to φ_d_ by the Boltzmann distribution as follows^18^:

1 $${\rm{\Delta }}{\phi }_{d}=\frac{kT}{e}\,\mathrm{ln}(\frac{{G}_{m}}{{G}_{m}^{0}}),$$

where *G*_*m*_ and *G*_*m*_^*0*^ are the steady-state membrane conductances induced by K^+^-nonactin in the presence and absence of the anesthetic, respectively, and *e*, *k*, and *T* have their standard meanings.

### Measurement of SRE channel cation/anion selectivity

The transport numbers of K^+^ (*t*^+^) and Cl^−^ (*t*^*−*^ = 1 − *t*^+^) was assessed by measuring zero current (reversal) potential (*V*^*rev*^) under 10-fold salt concentration gradient and by using the general expression^53^

2 $${V}^{rev}=(kT/e)(1-2{t}^{+})\mathrm{ln}({\gamma }_{1}{C}_{1}/{\gamma }_{2}{C}_{2}),$$

where *γ*_1_, *γ*_2_, *C*_1_ and *C*_2_ indicate activity coefficients and KCl concentrations in the *cis* and *trans* compartments, respectively. To measure the selectivity in the presence and absence of the drugs SRE treated membranes of similar macroscopic currents were tested.

### Computational optimization of the anesthetic molecules

Calculations of the geometric parameters of the LA molecules were performed by HyperChem 7.0 (Hypercube, Inc., Gainesville, FL, USA) using the semi-empirical method established by Hartree-Fock-Rutaan based on STO-3G. Using this method, the error in the bond lengths was 0.003 nm, and that in the angles was 4°. Furthermore, the local anesthetics were approximated as spheres with diameters corresponding to the maximum linear dimensions of the optimized molecules.

### Data availability

The datasets generated and analysed during the current study are available from the corresponding author on reasonable request.
